# Protein phosphatases regulate the liver microenvironment in the development of hepatocellular carcinoma

**DOI:** 10.1038/s12276-022-00883-0

**Published:** 2022-11-15

**Authors:** Joon-Sup Yoon, Chang-Woo Lee

**Affiliations:** 1 Department of Molecular Cell Biology, Samsung Medical Center, Sungkyunkwan University School of Medicine, Suwon, 16419 Republic of Korea; 2 Department of Health Sciences and Technology, SAIHST, Sungkyunkwan University, Seoul, 06351 Republic of Korea

**Keywords:** Cancer microenvironment, Post-translational modifications

## Abstract

The liver is a complicated heterogeneous organ composed of different cells. Parenchymal cells called hepatocytes and various nonparenchymal cells, including immune cells and stromal cells, are distributed in liver lobules with hepatic architecture. They interact with each other to compose the liver microenvironment and determine its characteristics. Although the liver microenvironment maintains liver homeostasis and function under healthy conditions, it also shows proinflammatory and profibrogenic characteristics that can induce the progression of hepatitis and hepatic fibrosis, eventually changing to a protumoral microenvironment that contributes to the development of hepatocellular carcinoma (HCC). According to recent studies, phosphatases are involved in liver diseases and HCC development by regulating protein phosphorylation in intracellular signaling pathways and changing the activities and characteristics of liver cells. Therefore, this review aims to highlight the importance of protein phosphatases in HCC development and in the regulation of the cellular components in the liver microenvironment and to show their significance as therapeutic targets.

## Liver cancer: Hitting the ‘off’ switch

Enzymes called phosphatases, which deactivate proteins by removing a phosphate group, may be an excellent therapeutic target for treatment of hepatocellular carinoma (HCC), the most common liver cancer. Most liver cells perform essential liver functions, such as metabolism and detoxification. The remaining cells regulate the liver microenvironment, responding to injury and controlling inflammation. Overactivation of immune cells following liver injury or disease can lead to chronic inflammation, which promotes tumor growth. Chang-Woo Lee and Joon-Sup Yoon at Sungkyunkwan University in Seoul, South Korea, have reviewed the roles of phosphatases in HCC. They report that regulating phosphatases can reduce risk of HCC development. Some phosphatase inhibitors may also boost the efficacy of anti-cancer drugs, and several are being tested in clinical trials. This review illuminates recent advances in understanding the mechanism and potential treatments for HCC.

## Introduction

The cellular components of the liver include hepatocytes, Kupffer cells, monocyte-derived macrophages (MoMФs), hepatic stellate cells (HSCs), T cells, and neutrophils. The arrangement of hepatic parenchymal and nonparenchymal cells determines hepatic architecture is developed and the cellular interactions in the liver microenvironment. Composed of hepatocytes, the hepatic parenchyma occupies ~75% of liver volume and performs most liver functions, such as metabolism, detoxification, and protein synthesis^1,2^. The hepatic nonparenchymal consists of immune cells (including Kupffer cells) and stromal cells [including liver sinusoidal endothelial cells (LSECs) and HSCs]. Although nonparenchymal cells are not significantly involved in the performance of a liver function, they contribute to the function and homeostasis of a healthy liver^3–6^. Nonparenchymal cells also sensitively respond to liver damage by secreting extracellular signaling molecules, such as cytokines, inducing the transition of the liver microenvironment, eventually leading to a proinflammatory microenvironment^6–8^. In the early stage of liver diseases, a proinflammatory immune response can regenerate homeostasis by promoting dead cell clearance, eliminating infection, and supporting the proliferation of stem cells. Chronic inflammation supports tumor growth by enhancing angiogenesis, reducing antitumor immunity, recruiting stromal cells, and causing the dedifferentiation of epithelial cells^9^. Ultimately, the proinflammatory liver microenvironment is converted into a protumoral microenvironment that promotes the development of liver tumors^9,10^.

Hepatocellular carcinoma (HCC) is a primary malignant liver tumor that accounts for most primary liver cancers^11^. Chronic hepatitis, which is a result of chronic hepatitis B, chronic hepatitis C, and nonalcoholic steatohepatitis (NASH), is an important risk factor for HCC. Notably, the annual incidence of HCC is significantly higher in patients with hepatitis-related cirrhosis than in patients with noncirrhotic chronic hepatitis^12^. In addition, cirrhosis is present in ~90% of patients with HCC. Collectively, these findings indicate that hepatic cirrhosis is the highest risk factor for HCC development. The development of HCC is closely associated with the liver microenvironment of hepatitis and hepatic cirrhosis. In a proinflammatory liver microenvironment, damage-associated molecular patterns (DAMPs) or pathogen-associated molecular patterns (PAMPs) derived from hepatocyte death or gut-derived microbes can stimulate Kupffer cells to secrete cytokines and chemokines, resulting in the recruitment of various immune cells and HSCs to the damaged sites^13–15^. Recruited HSCs are activated via the stimulation of interleukin-6 (IL-6), IL-17, and transforming growth factor-β1 (TGF-β1)^16–18^, inducing an imbalance of metalloproteinase (MMP)/tissue inhibitor of metalloproteinase (TIMP) and increasing the secretion of collagen fibers in the extracellular matrix (ECM), which leads to a profibrogenic liver microenvironment^19^. In a profibrogenic liver microenvironment, TGF-β induces the epithelial–mesenchymal transition (EMT) of hepatocytes to promote HCC progression^20^. Simultaneously, excess ECM deposition of collagen fiber induces hepatic cirrhosis^19^, which contributes to the development of a protumoral liver microenvironment with hypoxia^21^. Hypoxia enhances the development of a protumoral liver microenvironment by inducing tumor invasiveness, angiogenesis, and metastasis^22,23^. In the tumor microenvironment, increased C–C motif chemokine ligand 2 [CCL2, also known as monocyte chemoattractant protein-1 (MCP-1)] and IL-6 induce the recruitment of myeloid-derived suppressor cells (MDSCs), which are known to be immune-suppressive cells that inhibit antitumor immunity^24^. Thus, the liver microenvironment governs the development or potential regression of liver disorders, hepatitis, fibrosis, and HCC through the counteraction between acellular and cellular components of the liver microenvironment and their regulation by intercellular and intracellular signaling.

Posttranslational modification of proteins alters the functions of proteins and protein‒protein interactions by changing protein structures^25^. Phosphorylation and dephosphorylation are reversible posttranslational modifications of proteins that generally regulate the activation and inhibition of intracellular signaling pathways. More than 70% of cellular proteins have been shown to be regulated by phosphorylation and dephosphorylation^26^. Protein phosphorylation by kinases is a regulatory mechanism in various cellular activities and has been shown to be an important regulatory mechanism in HCC development and progression^27^. However, compared to kinases, fewer phosphatases, which regulate dephosphorylation, have been identified despite their importance. In humans, ~518 kinases have been identified, whereas only 137 phosphatases have been revealed^28,29^. Nonetheless, phosphatases are known to play critical roles in the development of liver diseases and HCC, as indicated by previous research. Studies have revealed that various phosphatases, including phosphatase and tensin homolog (PTEN), protein phosphatase 2 (PP2A), Src homology region 2 domain-containing protein tyrosine phosphatase-1 (SHP-1), and Src homology region 2 domain-containing protein tyrosine phosphatase-2 (SHP-2), contribute to the transition of characteristics of the liver microenvironment and HCC development by regulating cellular activities of parenchymal cells, stromal cells, and immune cells that compose the liver microenvironment^30–33^. These actions indicate that phosphatases can regulate HCC progression by determining the characteristics of the liver microenvironment via the regulation of intracellular signaling pathways in liver cells. This review highlights the importance of protein phosphatases in regulating the cellular components of the liver microenvironment and their significance as therapeutic targets.

## The transition of the liver microenvironment induces changes in HCC development

### Hepatic architecture

**Fig. 1:**
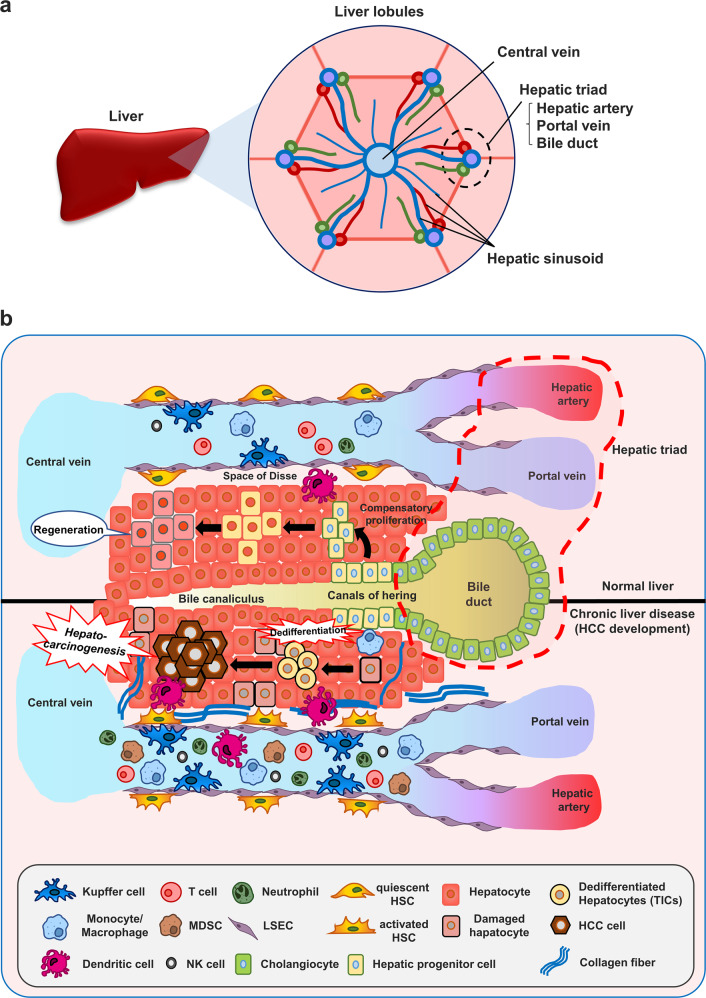
Model of liver regeneration or HCC development by hepatic progenitor cell expansion or hepatocyte dedifferentiation following liver injury/damage. **a** The liver consists of hexagonal liver lobules. **b** In the normal liver, regeneration occurs by compensatory proliferation of hepatic progenitor cells. However, in chronic liver diseases, dedifferentiation of mature hepatocytes can be induced. Tumor-initiating cells can promote the development of HCC through oncogenic proliferation.

The function and interaction of liver cells, including hepatocytes, immune cells, and stromal cells, are important for HCC development. Liver cells are functionally distributed on the basis of their hepatic architecture and thus compose the liver microenvironment. The liver lobule, the basic structure of the hepatic architecture, is a small hexagonal unit with hepatic sinusoids extending radially around the central vein, the terminal branch of the hepatic vein. The hepatic triad composed of the portal vein, hepatic artery, and bile duct is located at the point where liver lobules meet each other^34^ (Fig. 1a). Hepatic sinusoids that link blood flow from the portal vein and hepatic artery to the central vein are separated and are both surrounded by LSECs (Fig. 1b). Hepatocytes are arranged in the space between hepatic sinusoids. HSCs reside in the space of Disse^34^ (Fig. 1b). In the early stage of liver diseases, HSCs induce the recruitment of proinflammatory immune cells because of their special localization^35^. Through the hepatic sinusoidal lumen, most immune cells, including T cells, neutrophils, Natural killer (NK) cells, and monocytes, circulate through the liver with erythrocytes. Since Kupffer cells reside in the sinusoidal lumen, they can easily recognize liver injury, migrate to the injury site, recruit immune cells, and activate HSCs^34^. Bile canaliculi surrounded by hepatocytes transport generated bile to the bile duct. A stem cell niche known as the canal of hearing is located between cholangiocytes and hepatocytes^36^. In a healthy liver, hepatocyte death caused by damage is recovered by controlled compensatory proliferation that is induced by hepatic progenitor cells (also called facultative stem cells in humans and oval cells in rodents) in the canal of hering^36^ (Fig. 1b). However, chronic liver diseases lead to the dedifferentiation of mature hepatocytes and the transition of hepatocytes into tumor-initiating cells (TICs), through which uncontrolled oncogenic proliferation is mediated^37^ (Fig. 1b). Under these circumstances, a protumoral liver microenvironment can promote the development of HCC.

Although immune cells maintain liver homeostasis in a steady state, they can create a proinflammatory liver microenvironment by secreting proinflammatory cytokines and promoting the recruitment of inflammatory immune cells to sites of liver injury. Activated HSCs in a proinflammatory liver microenvironment can induce ECM deposition of collagen fiber and promote hepatic cirrhosis, which is a late-stage liver disease^19^. Excess ECM deposition can disrupt the hepatic architecture and induce capillarization of hepatic sinusoids, into which the fenestrae of LSECs shrink and disappear and a subcutaneous basement membrane is formed^38–40^. Hepatic sinusoidal capillarization reduces blood flow following increased vesicular resistance and causes hypoxia in the liver microenvironment^41^. Hypoxia induces angiogenesis and activates immune suppressive cells such as MDSCs and regulatory T cells (Tregs), contributing to a protumoral liver microenvironment^42^. Hence, the hepatic architecture plays a critical role in the function and interaction of liver cells. It is highly involved in HCC progression by transitioning the liver microenvironment.

### Hepatocytes

**Fig. 2:**
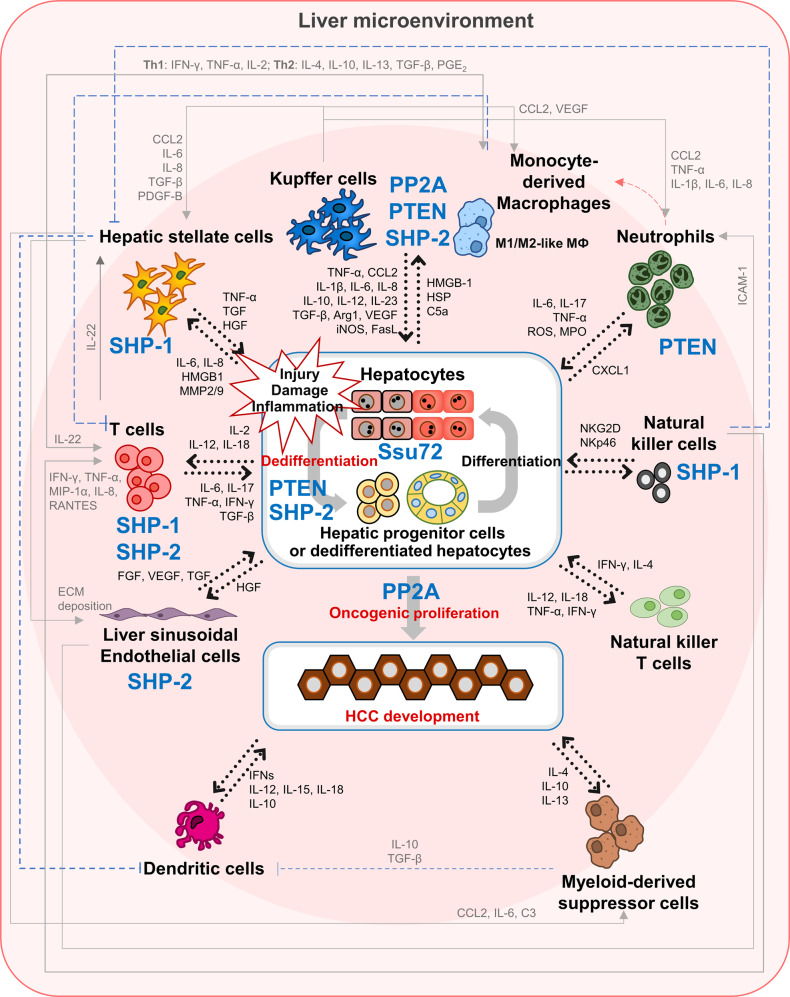
Phosphatases are associated with HCC development by regulating the development of a protumoral liver microenvironment. A detailed discussion of this model is provided in the text.

Hepatocytes are epithelial cells that organize the hepatic parenchyma and perform most liver functions. Since the liver is not only a primary organ for drug metabolism but is also directly linked with the gut, which supplies antigens, the liver is exposed to various risk factors that induce hepatocyte injury. Damaged hepatocytes can release DAMPs, activating Kupffer cells and HSCs and promoting the development of chronic hepatitis, which causes HCC development (Fig. 2). For instance, bacterial endotoxins, such as lipopolysaccharide (LPS) and an overdose of acetaminophen (APAP), can induce hepatocyte necrosis and release high mobility group box 1 (HMGB1), a DAMP, from necrotic hepatocytes^43,44^. Released HMGB1 can activate myeloid differentiation primary response gene 88 (MyD88)/nuclear factor-κB (NF-κB) through Toll-like receptor 4 (TLR4) signaling^45^. The apoptosis of damaged cells is important in preventing the rise of malignant cells, but NF-κB activation inhibits apoptosis and induces the proliferation of hepatocytes^46^. Moreover, NF-κB activation in hepatocytes induces IL-6-independent cytokine-induced neutrophil chemoattractant type-1 (CXCL1) production and the mobilization of neutrophils^47^ (Fig. 2). Recruited neutrophils resolve inflammation by eliminating pathogens and necrotic debris but enhance the inflammatory response by secreting proinflammatory cytokines, such as IL-1β, IL-6, and tumor necrosis factor-α (TNF-α)^48,49^. Secreted TNF-α encourages NF-κB activation, promoting the inhibition of apoptosis and increasing the proliferation rate of hepatocytes^46,49^. Thus, injury to hepatocytes concurrent with the inhibition of apoptosis invigorates a proinflammatory liver microenvironment and induces the conversion of hepatocytes into malignant cells.

A proinflammatory microenvironment leads to hepatic fibrosis/cirrhosis, which is accompanied by a profibrogenic liver microenvironment. In addition, hepatic cirrhosis induces hypoxia, resulting in a protumoral liver microenvironment. According to a recent study, hypoxia (3% O_2_) promotes reactive oxygen species (ROS)-induced EMT and increases the invasiveness of HCC cell lines^50^. HepaRG cells, showing characteristics similar to those of primary human hepatocytes, increase a master angiogenesis regulator hypoxia-inducible factor (HIF)-1α and cancer stem cell marker SRY-Box transcription factor 9 (SOX9) expression under hypoxia (5% O_2_)^51^. Consistent with the master regulator in hepatocytes, HNF4α expression is decreased in hepatocytes when the oxygen supply is lower than that in the blood circulatory system^52^. The decline in HNF4α causes dedifferentiation of hepatocytes, contributing to HCC development^37^. In addition, hypoxia induces the inverse of hepatic fibrosis. When mouse primary hepatocytes were exposed to 1% oxygen, the expression levels of myofibroblast marker genes, including alpha smooth muscle actin (α-SMA), Snail, and fibroblast-specific protein (FSP)-1, were upregulated^50^. However, hepatic cirrhosis increases TGF-β production in the liver microenvironment. TGF-β promotes the EMT and migration of hepatocytes, and the EMT of hepatocytes can increase the number of fibroblasts closely associated with tumor development, metastasis, and therapeutic resistance^53,54^. In addition, HIF-1α plays an important role in the TGF-β-mediated EMT of hepatocytes^50^. Therefore, a profibrogenic liver microenvironment promotes the EMT of hepatocytes that are induced by hypoxia and TGF-β signaling and lead to an increase in fibroblast generation, thereby faithfully fulfilling the role of an intermediate stem cell and contributing to the composition of a protumoral liver microenvironment and HCC development.

In liver diseases, proinflammatory cytokines and growth factors produced by immune cells and HSCs regulate the fate and proliferation of hepatocytes, inducing HCC development by activating various phosphorylation cascades. For instance, the mitogen-activated protein kinases (MAPKs), Janus kinase (JAK)/signal transducer and activator of transcription (STAT), phosphatidylinositol-3-kinase (PI3K)/Akt/mammalian target of rapamycin (mTOR), Wnt/β-catenin, NF-κB, and Ras signaling pathways are classical oncogenic pathways in HCC that promote HCC progression^55–61^. In mice, activation of Akt/mTOR and Ras/MAPK cascades results in rapid HCC development^62^. In a proinflammatory liver microenvironment, PI3K/Akt/mTOR cascades are activated by TNF-α, insulin-like growth factor (IGF), and hepatocyte growth factor (HGF), which are secreted by immune cells and HSCs^63–65^. Upregulation of PI3K/Akt activation inhibits apoptosis and maintains resistance against chemotherapy (docetaxel and sorafenib) in the Huh-7 HCC cell line^66^. Moreover, PI3K/Akt inhibition reverses the resistance to sorafenib in Huh-7 cells^67^. In addition, increased IL-6 levels in a proinflammatory liver microenvironment induce IL-6/JAK/STAT cascades, leading to HCC development via the accumulation of genetic alterations and enhanced STAT3 activity in hepatocytes^68^. In support of these findings, IL-17 has been shown to promote HCC progression by activating Akt-dependent IL-6/JAK2/STAT3 in HCC cells^69^. Indeed, phosphorylation of STAT3-induced marked increases in HCC generation, tumor number, and tumor size in mice^70^. Therefore, various phosphorylation cascades are activated by proinflammatory cytokines and enhance HCC progression by increasing the proliferation and survival of tumor cells. Collectively, liver diseases stimulate nonparenchymal cells, causing the transition of the liver microenvironment and increasing the potential for HCC development by disrupting the function of hepatocytes.

### Kupffer cells and monocyte-derived macrophages

Liver macrophages comprise Kupffer cells, which are nonmigratory liver-resident macrophages derived from the yolk sac or fetal liver, and MoMФs, which are differentiated from monocytes recruited by secreted chemokines. In the mouse liver, Kupffer cells are identified as F4/80^high^ cells, and CD11b^low^ cells and MoMФs are identified as F4/80^int^ and CD11b^high^ cells^71^. Kupffer cells and MoMФs comprise different subsets of liver macrophages, but they both play important roles in the resolution and progression of hepatitis and HCC development.

Kupffer cells are dominant liver-resident macrophages that regulate liver homeostasis in the normal liver. Since Kupffer cells reside in the hepatic sinusoidal lumen, they can rapidly respond to danger signals such as DAMPs or PAMPs and regulate the immune response and activation of HSCs^72^ (Fig. 1b). In the steady state, Kupffer cells govern the clearance of cell debris and microbes and maintain the function of a normal liver. However, excessive stimulation due to liver injury induces Kupffer cells to secrete cytokines, causing the transition of the liver microenvironment and inducing HSC activation. Kupffer cells sense DAMPs (such as HMGB1) and produce proinflammatory cytokines, including IL-1β, IL-6, IL-23, and TNF-α^43,45^, which are known to activate NF-κB signaling in hepatocytes and induce CXCL1 expression^47,73^. Together, IL-1β induces intracellular adhesion molecule-1 (ICAM-1) production in LSECs^74^. Increasing CXCL1 and ICAM-1 levels in the liver microenvironment increases the recruitment of neutrophils into the liver^47,74^ (Fig. 2). Thus, the response of Kupffer cells to liver injury promotes a proinflammatory liver microenvironment and initiates liver diseases. In contrast to Kupffer cells that reside in the liver in a normal state, monocytes, which differentiate into MoMФs, are recruited by CCL2 secreted from Kupffer cells and promote a further inflammatory response exacerbating a proinflammatory liver microenvironment^18,75,76^. Treatment with LPS, a representative PAMP molecule, and N-acetyl-galactosamine (GalN) markedly increased the infiltration of monocytes and neutrophils into the liver, whereas Kupffer cells show only slightly increased infiltration^71^. In addition, co-treatment with LPS and GalN promoted the expression of proinflammatory cytokines, including IFN-γ, IL-1β, IL-6, CCL2, CCL5, and TNF-α, in monocytes^71^. Moreover, differentiated THP-1 human monocytes show upregulated proinflammatory cytokines, including IL-6, IL-8, and TNF-α, due to an increase in the DNA-binding capacity of NF-κB, STAT3, and AP-1 after LPS treatment^77^. Moreover, LPS treatment potently induced IL-1β transcription and production in murine bone marrow-derived macrophages (BMDMs) mediated by prostaglandin E2 (PGE_2_)^78^. Therefore, increasing MoMФs in the early stage of liver diseases can be understood as a hallmark indicating proinflammatory progression of the liver microenvironment.

Upon microenvironmental stimuli, macrophages polarize that thus gain a proinflammatory/antitumoral or anti-inflammatory/protumoral phenotype. Kupffer cells can also be polarized into the classic proinflammatory M1 phenotype or alternative protumoral M2 type upon exposure to danger signals. M1 polarization leads to the secretion of IL-6, IL-12, TNF-α, and inducible NO synthase (iNOS) to promote proinflammatory responses against infection^79^, whereas M2 polarization of liver macrophages can promote liver fibrosis by expressing anti-inflammatory cytokines and profibrogenic mediators, such as IL-6, IL-10, macrophage colony-stimulating factor (M-CSF), arginase 1 (Arg1), and platelet-derived growth factor (PDGF)-B^80,81^. Although these proinflammatory cytokines enhance immune responses, IL-6 and TNF-α enhance survival signaling in neoplastic cells^82^. Proinflammatory responses result in the clearance of DAMPs and PAMPs from the liver microenvironment by inducing phagocytosis of hepatic phagocytes and inhibiting the death of hepatocytes^46,49,72^. HMGB1 released into the portal circulation promotes M1 polarization of Kupffer cells mediated by HMGB1/TLR4 signaling, whereas M2 polarization is increased following HMGB1 neutralization^83^. Although M2 polarization is associated with the resolution of inflammation and induces restoration of damaged liver the, M2-like function of Kupffer cells contributes to the development of liver fibrosis. For instance, Kupffer cell-derived TGF-β induces activation of HSCs in CCl_4_-induced liver fibrosis^84^. Activated HSCs also induce M2 polarization of MoMФs^35^. In addition, HSC activation can enhance a profibrogenic liver microenvironment, encouraging hypoxia. Hypoxia induced by cirrhosis can cause Kupffer cells to exert protumoral effects. Indeed, Kupffer cells activate HIF-1α and increase the expression levels of PDGF-B, vesicular endothelial growth factor (VEGF), angiopoietin-1, and CCL2 upon exposure to hypoxia (1% O_2_)^85^. Thus, throughout the progression of liver diseases, Kupffer cells contribute to the development of a protumoral liver microenvironment and eventually promote tumorigenesis in the liver. In the tumor microenvironment, anti-inflammatory M2 tumor-associated macrophages (TAMs) are associated with aggressive tumor progression, including tumor invasion and metastasis^86^. Moreover, although CCL2-increased extracellular signal-regulated kinase 1/2 (ERK1/2) phosphorylation is correlated with LPS/TLR signaling that results in inflammatory responses of MoMФs^87^, CCR2-positive proinflammatory M1 MoMФs undergo the M2 phenotypic transition in liver fibrosis mediated through the CCL2/CCR2 axis^35^. In addition, polarization to M2-like MoMФs is induced by Th2-derived cytokines such as IL-4, IL-10, IL-13, TGF-β, and PGE_2_^88,89^. Interestingly, CD11b^+^ F4/80^+^ M2-like MoMФs eliminated antigen-specific CD8^+^ T cells via the Fas/FasL pathway, induced immunosuppression, and diminished immunotherapy efficacy in the context of liver cancer metastasis^90^. Collectively, Kupffer cells and MoMФs closely interact with the liver microenvironment, and their functional changes contribute greatly to the transition of the liver microenvironment and the development of HCC.

### Hepatic stellate cells

HSCs are representative stromal cells of the liver that are strongly involved in hepatic fibrosis/cirrhosis. Because HSCs reside in the space of Disse, HSCs are directly activated by hepatocytes or Kupffer cell-derived signals in a proinflammatory liver microenvironment (Fig. 1b). After activation, HSCs induce the transition of immune cell function and composition, contributing greatly to the development of a profibrogenic/protumoral liver microenvironment. To examine how HSCs regulate the liver microenvironment, Robert et al. compared the effects of proinflammatory cytokines, including IL-1α/β, IL-8, and TNF-α, and the profibrogenic mediator TGF-β in LX-2, a human HSC cell line^19^. As expected, TGF-β treatment induced the downregulation of MMP-1 and MMP-3 but induced the upregulation of profibrogenic proteins such as TIMP-1, collagen type I/IV, α-SMA, hydroxyproline, and PDGF-B^19,84^. In addition, the levels of most proinflammatory cytokines and chemokines, except IL-6, were reduced in LX-2 cells^19^. As a result of HSC activation, the liver microenvironment presents profibrogenic features, and liver diseases progress to hepatic fibrosis/cirrhosis under hypoxic conditions. In contrast, hypoxia due to hepatic fibrosis/cirrhosis is important to the function and activation of HSCs. Exposure to hypoxia (0.5% O_2_) activates HIF-1/2α in HSCs^91^. Activated HIF-1/2α positively regulates the expression of genes that are important for HSC function, angiogenesis, and collagen synthesis in HSCs^91^. Furthermore, activated HSCs induce not only cause hepatic fibrogenesis but also an immunosuppressive liver microenvironment, promoting the development of a protumoral liver microenvironment. Activated HSCs secrete CCL2 to increase the recruitment and infiltration of CCR2^+^ MoMФs, which first polarize into the proinflammatory M1 phenotype but eventually polarize to the M2-like phenotype^35,36^. The recruitment of proinflammatory M1 macrophages and their functional transition into anti-inflammatory M2 macrophages mediated by HSCs is closely associated with the progression of liver disease into hepatic cirrhosis and the transition of the liver microenvironment into a protumoral environment. Moreover, HSCs can suppress dendritic cell (DC) propagation and promote the propagation of CD11b^+^CD11c^−^ cells from bone marrow-derived monocytes^92^. When monocytes are cocultured with HSCs, monocytes show lower levels of major histocompatibility complex class II (MHC class II), CD40, and CD86, a relatively higher level of F4/80 and significantly higher levels of programmed death-ligand 1 (PD-L1) and Gr-1 than are found in mature DCs^92^. Low levels of MHC class II, CD40, and CD86 imply an immature stage of DCs^92^. Immature DCs are less effective than mature DCs in inducing T-cell proliferation. These factors induce Treg activation by producing high levels of IL-10, an immunosuppressive cytokine^92,93^. Since mature DCs can secrete a variety of cytokines, including IL-12, IL-18, TNF-α, and IFN-γ, which act on natural killer T (NKT) cells and induce Th1 and CD8^+^ cytotoxic T cell activation, the propagation of immature DCs promotes HCC progression^94^ (Fig. 2). Moreover, HSC-derived IL-6 and complement complex 3 (C3) induced bone marrow cells to differentiate into MDSCs and promoted HCC progression after orthotopic transplantation of HCC cells in mice^92,93^ (Fig. 2). Therefore, activation of HSCs is a key factor for the transition of the liver microenvironment into an immunosuppressive environment and the progression of hepatic fibrosis/cirrhosis, with both outcomes promoting HCC development.

Quiescent HSCs regulate the level of vitamin A in storage and in microcirculation in the normal liver^95^, but a proinflammatory liver microenvironment induces activation of HSCs, promoting hepatic fibrogenesis. The phosphorylation in signaling pathways is strongly associated with the activation of HSCs. For instance, IL-6 directly stimulates the activation of HSCs and induces the phenotypic transition of quiescent HSCs into myofibroblast-like cells by activating the JAK/STAT and MAPK signaling pathways^57^. In addition, IL-17 and IL-22 secreted by T helper 17 (Th17) cells activate HSCs and induce the expression of TGF-β by enhancing p38 MAPK signaling^96^ (Fig. 2). Oxidative stress caused by hepatic inflammation leads to liver fibrosis by activating apoptosis signal-regulating kinase 1 (ASK1)/MAPK signaling in HSCs^97^. In addition to cytokines, various danger signals in the inflammatory microenvironment lead to HSCs activation. HMGB1 can activate ERK/c-Jun and PI3K/Akt signals in HSCs via the receptor for advanced glycation end products (RAGE) receptor and induce the production of collagen type I^45^. Furthermore, activation of TLR4/MyD88/NF-κB signaling induced by LPS results in the downregulation of BMP and activin membrane-bound inhibitor (BMABI), a pseudoreceptor of TGF-β, and enhances the TGF-β-induced profibrogenic function of HSCs^98^. Notably, TGF-β is necessary to induce the activation of HSCs. TGF-β induces HSC activation mediated by Smad and MAPK signaling^99,100^. In particular, c-Jun N-terminal kinase 1 (JNK1), but not JNK2, has been shown to play an important role in promoting hepatic fibrogenesis^101^. Regarding the treatment of hepatic fibrosis, it has been shown that JNK inhibitors downregulate TGF-β and PDGF expression, affecting the activation of mouse primary HSCs and downregulating TGF-β and PDGF expression in human HSCs^101^. Therefore, suppressing the phosphorylation in signaling pathways related to HSC activation may be an efficient hepatic cirrhosis treatment and reduce the potential risk of HCC development.

### T cells

As central components of adaptive immunity, T cells contribute to the development and progression of hepatitis and HCC by producing various cytokines with cytotoxic functions. T cells are classified into CD4^+^ T helper cells and CD8^+^ cytotoxic T cells. Naïve CD4^+^ T cells can differentiate into Th1, Th2, Th17 cells, or Tregs. Th1 cells can secrete proinflammatory cytokines, including IFN-γ, TNF-α, and IL-2, enhance the motility and killing capacity of CD8^+^ cytotoxic T cells, and induce the M1 polarization of macrophages^102–104^ (Fig. 2). Although these functions of Th1 cytokines are associated with hepatitis progression^105^, Th1 cells can suppress HCC development by upregulating antitumor immunity^94^. Indeed, the number of Th1 cells and Th1 cytokines in hepatitis B virus (HBV)-related or hepatitis C virus (HCV)-related HCC was found to be lower than that in normal liver^94,106^. However, Th2 cells release the cytokines IL-4, IL-6, IL-10, and IL-13, which stimulate M2 TAMs, inducing tumor promotion and downregulating antitumor immunity^94,107^ (Fig. 2). Moreover, the expression levels of GATA-binding protein 3 (GATA3) and IL-4, which are Th2 target genes, have been positively correlated with the levels of immune checkpoint proteins [programmed cell death-ligand 1 (PD-L1), PD-L2, and PD-1] in patients with cancer^108^. Furthermore, Th2 dominance has been associated with HCC pathogenesis of HCV-related liver cirrhosis^94^. Th17 cells mediate pathogen clearance, inflammation, and autoimmune diseases. Recently, the frequency of splenic Th17 cells in nonalcoholic fatty liver disease (NAFLD)-induced mice was reported to be increased^109^. Th17 cells are the primary sources of IL-17 and IL-22, which are involved in hepatitis, cirrhosis, and HCC progression^109^. IL-17 significantly promotes the invasion and wound-healing capacity of HCC cells following the upregulation of IL-6, IL-8, MMP-2, MMP-9, and VEGF^69,110^. Similarly, the high frequency of IL-17^+^ cells has been positively correlated with HCC metastasis, overall survival (OS), and the disease-free survival (DFS) rate^110^. In support of these findings, high expression levels of intratumoral IL-17 and IL-17RE have been found to be closely associated with the poor prognosis of HCC^111^. Moreover, IL-17RA is expressed in HCC cells, such as Huh-7 and SMMC7721 cells^69^.

T-cell-mediated immune responses play important roles in the liver microenvironment and HCC progression. In particular, the ratio of T helper cell subsets can regulate HCC progression. An imbalance in Th1/Th2 (that is, a low number of Th1 cells and a high number of Th2 cells) is associated with poor prognosis of cancer patients^112^. However, the ratio of Th17/Th1 is higher in HBV-related HCC patients than in patients with non-HBV-related HCC. Intratumoral and peritumoral Th17 counts show a reverse correlation with OS and DFS rates^106^. Therefore, a high Treg/Th17 ratio is related to HCC pathogenesis. It is also associated with poor prognosis in HCC patients^113^. Thus, the balance of T helper cell subsets can be considered a prognostic marker for the progression of liver diseases.

### Neutrophills

Neutrophils are the most common effector cells of innate immunity in mammals. As the first line of innate immunity, neutrophils are rapidly recruited into tissues via the circulatory system. They play an especially important role in bacterial and viral infections in tissues. They exhibit invading pathogen-eliminating and cytotoxic functions by releasing neutrophil extracellular traps (NETs), leading to ROS production, cytokine and chemokine secretion, degranulation, and phagocytosis^49^. Moreover, neutrophils play crucial roles in mediating inflammation and immune responses. In the early phase of liver diseases or injury, neutrophils are the first immune cells to migrate into the liver, and their responses determine whether additional inflammatory responses should be promoted or suppressed^114^. For instance, CD66^+^ neutrophils are the main sources of the proinflammatory cytokine IL-17 in the liver^96^. On the other hand, recruited neutrophils, owing to acute liver inflammation caused by, for example, an overdose of APAP, execute immunosuppressive functions by mediating the development of anti-inflammatory or reparative Ly6c^low^ CX3CR1^high^ macrophages^115^. Neutrophils not only can mediate inflammatory responses but can also accelerate liver fibrosis in chronic hepatitis. In viral hepatitis, neutrophils express proinflammatory cytokines, such as IL-6 and TNF-α following activation of TLR8, which can recognize single-stranded viral RNA^116^. However, chronic hepatitis B can induce dysfunction of neutrophils, as indicated by a decrease in the release of NETs^111,117^. The dysfunction of neutrophils is induced by the HBV core protein and HBV envelope protein, which inhibit the ERK1/2, P38 MAPK, and mTOR pathways, thereby suppressing ROS production in neutrophils^111^. Since it may lead to failed elimination of pathogens from the liver, neutrophil dysfunction increases complications, morbidity, and mortality in hepatic cirrhosis patients^117,118^. Thus, neutrophils can induce the progression of liver diseases and hepatic fibrosis and at the same time exhibit a bipotential function by protecting the liver from infection.

Moreover, dysfunctional neutrophils can promote the development of hepatic cirrhosis under hypoxic conditions, which is key to a protumoral liver microenvironment. Hypoxia inhibits the apoptosis of neutrophils, disrupting the mechanism that regulates neutrophil functional longevity^119^, and the tumoricidal capacity of neutrophils can be suppressed by hypoxia^120^. For instance, surviving neutrophils in hypoxic conditions can contribute to the migration and invasion of cancer cells by expressing MMP-8 and MMP-9^121,122^. In addition, a recent study showed that NET formation promoted the metastasis of HCC^123^. Trapped HCC cells in NETs induced resistance to cell death and enhanced their invasiveness by activating TLR4/9-cyclooxygenase 2 (COX-2) signaling^123^. Similarly, neutrophils obtained from HCC patients show enhanced NET formation capacity^123^. Thus, neutrophils can inhibit the progression of liver diseases and HCC, but they may lose their function and contribute to disease progression via the influence of the liver microenvironment.

### Natural killer cells

NK cells are effector cells of innate immunity. They maintain immune surveillance and exert cytolytic functions against physiologically stressed cells, such as tumor cells and virus-infected cells^124^. NK cells exhibit antiviral functions not only by promoting IFN-γ and TNF-α production but also by inducing cytotoxicity^125^. However, in chronic hepatitis B patients, NK cells become dysfunctional because of impaired mTOR signaling^126^. This dysfunction can promote the progression from hepatitis to hepatic fibrosis. On the other hand, NK cells play a paradoxical role in hepatic fibrosis. NK cells inhibited hepatic fibrosis by killing early activated HSCs through retinoic acid early inducible 1 (RAE-1) ligand and natural killer Group 2D (NKG2D) receptor activity in mice^127,128^ (Fig. 2).

NK cells occupy crucial positions in tumor surveillance. Activation of NK cells is triggered in many ways, including the lack of MHC class I molecules on tumor cells and the recognition of stress-induced molecules by NK-cell-activating receptors (NKG2D and NKp46)^124,127^. Activated NK cells secrete IFN-γ, TNF-α, macrophage inflammatory protein 1α (MIP-1α), and IL-8 and regulate activation; normal T cells express and secrete [RANTES, also known as C–C motif chemokine ligand 5 (CCL5)], which can induce the migration of T cells^129,130^. In addition, NK cells activated by IL-12/15/18 can kill HCC cells regardless of NKG2D expression^131^. However, in the late stage of HCC, tumors form a barrier and interrupt the infiltration of NK cells into the inside of an HCC tumor. Thus, NK cells become exhausted due to the decrease in an activating marker^132^. This NK cell exhaustion is a critical problem for the treatment of HCC. NK-cell activity can affect the recurrence rate in HCC patients. Patients with a low proportion of INF-γ-producing NK-cells (<45%) show a significantly higher HCC recurrence rate than patients with a high IFN-γ-producing NK-cell proportion (≥45%) one month after treatment^133^. Collectively, NK cells play central roles in inhibiting the development and progression of HCC and killing of tumor cells, but they can be exhausted by the transition of the liver microenvironment and thus not function efficiently.

## Protein phosphatases are involved in the development of HCC

Various cellular activities, including immune responses, the maintenance of cellular function, and cell activation, proliferation, and dedifferentiation in the liver microenvironment of patients with HCC, are regulated by intracellular phosphorylation in signaling pathways that can dynamically respond to stimulation in the extracellular environment. Therefore, phosphatases play important roles in the transition of the liver microenvironment to regulate HCC development.

### PTEN

PTEN is a well-characterized tumor suppressor gene that is commonly inactivated in human cancers. PTEN suppresses HCC development by preventing the proliferation of hepatocytes through Akt/mTOR inactivation^31,32^ (Fig. 2). In support of this finding, reduced or absent expression of PTEN has been observed in approximately one-half of hepatoma patients^134,135^. Moreover, hepatic PTEN is associated with NASH and NASH-associated HCC development. Specifically, mice with liver-specific PTEN deletion (Alb-Cre; Pten^flox/flox^) showed human NASH-like phenotypes such as hepatomegaly and steatohepatitis with triglyceride (TG) accumulation^134^. In this case, hepatocytes showed induction of adipocyte-specific genes and genes associated with lipogenesis and β-oxidation^134^. In addition, PTEN-deficient hepatocytes showed hyperproliferation, increased hydrogen peroxide, and abnormal activation of protein kinase B (PKB)/Akt and MAPK (ERK1/2), which many have resulted in hepatic tumorigenesis^134^. Indeed, all PTEN-deficient mice presented with liver tumors, with 66% of the classified as HCC^134^. In line with these results, another study observed that PTEN deficiency along with loss of SHP-2 promoted NASH development and TIC generation^136^. PTEN deficiency-induced Akt activation and SHP2 deficiency-induced JNK activation resulted in the increased expression and activation of c-Jun, which promotes HCC development^136^. Moreover, PTEN reduction has been associated with increased expression of cancer stem cell markers [such as CD133, epithelial cell adhesion molecule (EpCAM), and CK19], HCC prognosis, and HCC recurrence^135^. These findings suggest that the PTEN/Akt/mTOR pathway regulates malignant hepatic tumorigenesis and affects HCC recurrence and overall survival by regulating cancer stem cells^135^. Collectively, these findings indicate that PTEN plays important roles in regulating the homeostasis and metabolism of hepatocytes and in suppressing HCC development.

PTEN is critical for regulating macrophage polarization and function in the tumor microenvironment (Fig. 2). N-myc downstream-regulated gene 2 (NDRG2) recruits PP2A and regulates the activity of PTEN through the dephosphorylation of Ser380, Thr382, and Thr383 in the c-tail of PTEN^137^ (Fig. 2). It has also been speculated that PTEN might be involved in JAK/STAT and NF-κB signaling^137^. Furthermore, loss of NDRG2 in BMDMs enhanced IκB kinases α/β (IKKα/β), p65, and IκBα phosphorylation following Akt activation, leading to a polarization of M2 macrophages to an M1-like phenotype^33^. Collectively, PTEN dephosphorylation and activation via the NDRG2–PP2A complex promote cancer progression by increasing the number of M2 TAMs following the inhibition of NF-κB and IκB phosphorylation. On the other hand, the downregulation of PTEN in Raw264.7 cells remarkably increased the levels of CCL2 and VEGF-A, which are known to induce M2 macrophage polarization^80^.

### PP2A

PP2A is a serine/threonine dual-specific protein phosphatase with a tumor-suppressive function. PP2A is involved in various intracellular signaling pathways and processes in mammalian cells; it is involved in apoptosis, cell cycle, cell proliferation, cell migration, cell transformation, and transcription^138^. The function of PP2A as a tumor suppressor has been observed in HCC^139–141^. The activation of PP2A inactivated PP2A substrates, including β-catenin, c-Myc, and p-B-cell lymphoma 2 (Bcl-2), via its dephosphorylation, inducing an increase in the apoptosis and a decrease in the proliferation of HCC cell lines^139^. Moreover, activation of PP2A inhibited the increase in proliferating cell nuclear antigen (PCNA)-positive hepatocytes after DEN administration in vivo^139^. PP2A is involved in cancer metastasis. Suppression of PP2A via overexpression of an inhibitor of protein phosphatase 2A (CIP2A) or okadaic acid can induce the migration of HCC cells by increasing the expression of MMP-9 and TIMP-1, which causes the breakdown of the ECM^142^. PP2A also promotes cell transformation by interacting with simian virus 40 small t (SV40ST), an oncoprotein^141^. Inhibition of PP2A by SV40ST induces tumorigenic phenotype acquisition in human HEK TER cells by activating the PI3K/Akt, Wnt/β-catenin, and c-Myc pathways^141^. In addition, PP2A is involved in the sensitivity and activity of chemotherapy against HCC^140,143^. Interestingly, the transcription of PP2A-B55δ, a PP2A subunit, is decreased in HCC cell lines such as the HepG2, MHCC97H, MHCC97 L, Hep3B, and Huh-7 cell lines^140^. However, the administration of cisplatin (cDDP), a chemotherapy drug, leads to PP2A-B55δ expression^140^. PP2A-B55δ not only increases the tumor inhibitory effects of cDDP on cell migration, colony formation, proliferation, the cell cycle, and apoptosis but also increases its therapeutic effect^140^. On the other hand, it has been revealed that PP2A enhances the anticancer effect of erlotinib and bortezomib^143^. CIP2A inhibition mediates the apoptotic effect of TD52, an erlotinib derivative, via p-Akt downregulation caused by PP2A in HCC cell lines^143^. Collectively, PP2A can execute an antitumor function in hepatocytes of HCC (Fig. 2).

### SHP-1 (PTPN6)

SHP-1, encoded by the tyrosine-protein phosphatase nonreceptor type 6 (PTPN6) gene, is a cytoplasmic protein tyrosine phosphatase. SHP-1 plays a critical role in liver diseases by regulating the function of various nonparenchymal cells. SHP-1 plays a crucial role in innate immune cells. For instance, SHP-1 downregulation in macrophages can increase CCL2 expression, inducing the recruitment of immune cells in the proinflammatory liver microenvironment. Upon LPS administration, SHP-1 in macrophages enhanced the synthesis of IL-12p40, which is a common subunit of proinflammatory cytokines such as IL-12p70 and IL-23, by regulating TLR-induced PI3K/Akt activation, IκB degradation, and nucleosome remodeling^144^. In addition, loss of SHP-1 increased M2 phenotype acquisition (F4/80^+^, CD11b^+^, CD11c^−^) by macrophages in insulin-resistant mice with diet-induced obesity^145^ (Fig. 2). In contrast its involvement in proinflammatory functions in macrophages, SHP-1 can contribute to tumor development by inhibiting NK-cell activation. By causing the dephosphorylation of immunoreceptor tyrosine-based activation motifs (ITAMs) downstream via the interaction of killer cell Ig-like receptors (KIRs) with MHC class I molecules on target cells, which activates NK cells, SHP-1 can block NK-cell activation^146^.

Furthermore, SHP-1 plays important roles in T-cell development by regulating activation signaling pathways (Fig. 2). Casitas-B-lineage lymphoma (Cbl)-b, which is a downstream target of SHP-1, induces the ubiquitination of phospholipase C γ1 (PLC-γ1), regulating tolerance and the development of T cells^147,148^. SHP-1 regulates the degradation of Cbl-b by dephosphorylating its tyrosine residue^149^. To examine the effect of SHP-1 on T-cell development, T-cell-specific SHP-1-deficient mice were generated^149^. The T-cell-specific SHP-1-deficient mice showed aberrant increases in Th2 cell responses mediated by reduced Cbl-b expression^149^. In addition, another study revealed that loss of SHP-1 led to STAT6 phosphorylation maintenance and induced Th2 cell development^150^. Therefore, SHP-1 downregulation in T cells results in Th2 cell development and can lead to a poor prognosis for tumors.

T-cell receptor (TCR) stimulation is essential for T-cell activation, development, and differentiation. SHP-1 regulates tyrosine phosphorylation of zeta-chain-associated protein kinase 70 (ZAP-70), which is a key downstream regulator of TCR signaling^151^. Since tyrosine phosphorylation is essential for the activation of ZAP-70, SHP-1 can regulate TCR signaling and T-cell activation through the dephosphorylation of ZAP-70^151^. In T cells, the core protein of HCV inhibits SHP-1 expression, inducing hyperphosphorylation of ZAP-70, a linker for the activation of T cells (LAT), and PLC-γ, which are targets of SHP-1, without stimulating the CD3 receptor^152^. These studies imply that the downregulation of SHP-1 can lead to positive ZAP-70-dependent TCR signaling effects. In contrast, SHP-1 can suppress T-cell activation and promote the progression of infection. For instance, SHP-1 inhibited ERK2 activation and transcriptional activation of the IL-2 gene in T cells^153^. In addition, SHP-1 inhibited murine Th17 cell development by negatively regulating IL-6- and IL-21-dependent STAT3 phosphorylation^154^. Collectively, SHP-1 is directly involved in T-cell development as well as activation mediated by TCR signaling and can regulate disease progression in the liver microenvironment.

SHP-1 is involved in hepatic fibrogenesis by regulating the function of HSCs (Fig. 2). For instance, SHP-1 shows a tendency to increase in patients with chronic hepatitis B and advanced fibrosis^30^. SHP-1 activation decreased HSC proliferation and inhibited p-STAT3 to promote HSC apoptosis^30^. In particular, SHP-1 inhibits the activation and proliferation of HSCs by regulating p-ERK1/2, p-Akt, and p-PDGFR signaling^155^.

### SHP-2 (PTPN11)

Activating mutation of SHP-2, encoded by the protein tyrosine phosphatase nonreceptor type 11 (PTPN11) gene, has been observed in various cancers. SHP-2 is involved in cell survival and proliferation via RAS–ERK signaling pathway activation^156^. For instance, inhibition of SHP-2 suppressed the proliferation of receptor-tyrosine-kinase-driven human cancer cells through the RAS–ERK pathway^156^. Furthermore, Myc-derived oncogenesis depends on the RAS–ERK pathway, which is regulated by SHP-2, which maintains Myc stability^157^. In contrast, SHP-2 is a key mediator of immune checkpoint receptors such as PD-1 and B- and T-lymphocyte attenuator (BTLA)^156^ and plays an important role in antitumor immunity (Fig. 2). SHP-2 deletion induced the establishment of an immunosuppressive environment with defective TICs, aggressive tumor progression, and upregulated Wnt/β-catenin signaling^157^. These findings suggested that the downregulation of SHP-2 attenuates antitumor immunity. Indeed, a recent study found that SHP-2-deficient livers showed reduced M1 polarization, indicating phagocyte activity against Myc^+^ TICs^157^. In addition, loss of SHP-2 in the liver has been associated with increases in macrophage migration inhibitory factor (MIF), leukocyte cell-derived chemotaxin-2 (Lect2), and CCL17, which are expressed by noninflammatory macrophages and target Tregs^157^. In addition, low SHP-2 expression in TAMs can lead to a poor prognosis in patients with colorectal cancer^81^. It has been revealed that low SHP-2 expression in TAMs is associated with a high incidence of liver metastasis^81^. Consistently, loss of SHP-2 in BMDMs induces decreases in M1 macrophage-related gene expression (iNOS, IL-6, and TNF-α) and increases in M2 macrophage-related gene expression [Arg1, Fizz1, Ym-1, and IL-10] to promote M2 polarization of TAMs in the tumor microenvironment^81^. Collectively, SHP-2 can promote the development of HCC by their involvement in the proliferation and survival of tumor cells, but inhibition of SHP-2 in immune cells can suppress antitumor immunity. This duality of SHP-2 should be carefully considered when selecting SHP-2 as a therapeutic target.

### Ssu72

Ssu72 is a dual-specific protein phosphatase with serine/threonine or tyrosine residue activity. Ssu72 recognizes p-Ser5 and p-Ser7 in the C-terminal domain of RNA polymerase II and regulates transcription factor recruitment via hypophosphorylation of RNA polymerase II^158,159^. Our recent studies revealed that Ssu72 can dephosphorylate transcription factors in hepatocytes and regulate the cell cycle, homeostasis, and differentiation of hepatocytes^37,160^. In addition, loss of hepatic Ssu72 expression can induce liver injury and chronic liver diseases. It is also closely associated with HCC development through the dedifferentiation of hepatocytes.

We found that liver-specific Ssu72 depletion increased hepatic chromosome polyploidization and liver injury, presented as increases in fat storage, necrotic hepatocytes, inflammatory cell infiltration, cytoplasmic vacuolation, and fibrogenic cytokines (IL-6 and TNF-α)^160^. Moreover, depletion of hepatic Ssu72 resulted in a high incidence of NAFLD with lipid accumulation, ballooning hepatocytes, α-SMA upregulation, and increased proinflammatory cytokines (IL-1β, IL-6, and TNF-α)^160^. Although hepatocytes show the potential for increased proliferation, such as through increased expression of Ki-67 and PCNA, we did not observe the development of HCC in the Ssu72-depleted liver^160^. Importantly, we found that hepatic Ssu72 deletion upregulated cell cycle-related genes known to be associated with cell cycle control, DNA replication, cell proliferation, and DNA repair and is directly governed by the retinoblastoma protein (Rb)–E2F signaling pathway^160^. In addition, hepatic Ssu72 depletion caused hyperphosphorylation of Rb associated with entry into the cell cycle, interaction with E2F transcription factor 1 (E2F1), and activation of transcriptional regulation of E2F1 signaling, conferring a growth advantage onto quiescent cells^160^. Moreover, increased Ssu72 expression induced a decline in the transcriptional activity of E2F1^160^. Collectively, these results indicated that Ssu72 can regulate the transcriptional activity of E2F1 through the dephosphorylation of Rb in hepatocytes. Therefore, loss of hepatic Ssu72 can aberrantly activate the cell cycle by overriding restriction checkpoints and induce excessive mononucleated polyploid hepatocyte proliferation, contributing to the pathogenesis of chronic liver diseases (Fig. 2).

Although hepatocyte-specific Ssu72-depleted mice fed a normal chow diet showed no HCC development^160^, human patients with chronic liver diseases and NASH-associated HCC showed significantly downregulated Ssu72 expression in their livers^37^. Interestingly, liver-specific Ssu72-depleted mice showed markedly increased HCC development after administration of DEN, streptozotocin, or 3,5-diethoxycarbonyl-1,4-dihydrocollidine (DDC)^37^. Upon liver damage, Ssu72-depleted livers showed an increase in oval-shaped cells that expressed PCNA and upregulated expression of cancer stem cell markers such as EpCAM, Sox9, CD13, and CD133 near the pericentral area^37^. The increase in proliferating oval-shaped cells around the pericentral area is an abnormal finding and may contribute to HCC pathogenesis. Chronic hepatic damage causes the transition of hepatocytes into hepatic progenitor cells in the Ssu72-depleted livers^37^. Although the EMT or the dedifferentiation of hepatocytes into progenitor cells is a major mechanism of TIC generation in the pathogenesis of HCC, the molecular mechanism involved in reprogramming differentiated hepatocytes into dedifferentiated cells has not yet been discovered. Importantly, a positive correlation between the mRNA expression levels of Ssu72 and hepatocyte nuclear factor α (HNF4α), a master regulator of hepatocyte function, have been identified in the liver of NASH-associated HCC patients^37^. In addition, we found that hepatic Ssu72 depletion induced the downregulation of HNF4α target gene expression when the liver is damaged^37^. Hyperphosphorylation of HNF4α has been observed in Ssu72-depleted hepatocytes after DEN administration^37^. Ssu72 can physically bind to hyperphosphorylated HNF4α and regulate the transcriptional activity of HNF4α by mediating the dephosphorylation of p-Ser313, an inhibitory phosphorylated residue^37^. Collectively, these findings indicated that loss of hepatic Ssu72 under chronic liver disease conditions can induce hepatocyte-to-progenitor cell conversion, which is regulated by the inhibitory phosphorylation of HNF4α^37^ (Fig. 2).

## Clinical trials with phosphatase inhibitors

Alteration of phosphorylation in signaling pathways is considered one of the most critical contributors to the tumorigenesis of solid tumors, including HCC. These signaling pathways have received increasing attention as therapeutic targets due to their ability to regulate the cell cycle, survival, growth, and proliferation in tumor cells. For instance, the PI3K/AKT/mTOR oncogenic signaling pathway can regulate the cell cycle, cell growth, and cell proliferation^161^. This pathway is generally activated in cancers^162^. Importantly, SHP-2 can promote cell proliferation by activating the PI3K/AKT pathway^163,164^. Recently, phosphatases, which regulate kinase and phosphorylation signaling, have been suggested as novel therapeutic targets. Various clinical trials targeting phosphatases are underway for patients with advanced solid tumors.

PP2A is a tumor suppressor. PP2A inhibition is closely associated with the progression of cancer. Indeed, certain human cancers (40% of non-small cell lung carcinoma and 60% of prostate cancer) show inhibition of PP2A because of increases in PP2A inhibitors such as CIP2A and SET proteins^165^. Notably, tumor cells that show decreased PP2A activity are vulnerable to additional PP2A inhibition^165^. LB-100 is a PP2A inhibitor that reduces PP2A protein abundance in tumor cells and induces cell cycle progression without triggering a DNA damage repair response^166^. Thus, conventional anticancer agents designed to kill cells during the dividing process can more effectively kill tumor cells^166^. According to a clinical trial, NCT01837667, when PP2A activity was suppressed by LB-100, the therapeutic efficiencies of conventional anticancer agents, including temozolomide, docetaxel, doxorubicin, and cisplatin, were significantly increased without causing additional cytotoxicity^165^. In another trial, NCT01837667, Chung et al. tested the safety, tolerability, and preliminary activity of LB-100^167^. After LB-100 was injected daily for 3 days in 21-day cycles, it was revealed that among 27 participants, 6 participants (20.7%) showed drug-related grade three adverse events, and 20 patients were response evaluable^167^. Among these response-evaluable participants, 10 (50%) showed stable disease through four or more cycles, and one patient showed a partial response through five cycles^167^. Clinical trials using LB-100 against solid tumors are ongoing. A phase II trial (NCT03027388) designed to determine the therapeutic effect of LB-100 on recurrent glioblastoma is ongoing. Especially, the purpose of this Phase II trial was to examine whether LB-100 exerts its effect by crossing the blood‒brain barrier. Moreover, a Phase Ib trial (NCT04560972) that combines LB-100 with carboplatin, etoposide, or atezolizumab for treating previously untreated extensive-stage small-cell lung carcinoma is ongoing. The side effects and optimal dose of LB-100 are also being tested. In addition, several clinical trials (JAB-3068; NCT03565003, NCT03518554, NCT04721223, and ERAS-601; and NCT04670679 and NCT04959981) are underway to confirm the safety and tolerability of escalating doses of the SHP-2 inhibitors JAB-3068 and ERAS-601 in advanced or metastatic solid tumors. Collectively, the development of phosphatase inhibitors for treating advanced solid tumors has been clearly progressing recently. Many studies have attempted to improve the safety, tolerability, and therapeutic effects of phosphatase inhibitors through their coadministration with conventional anticancer agents.

## Therapeutic insights and conclusions

**Fig. 3:**
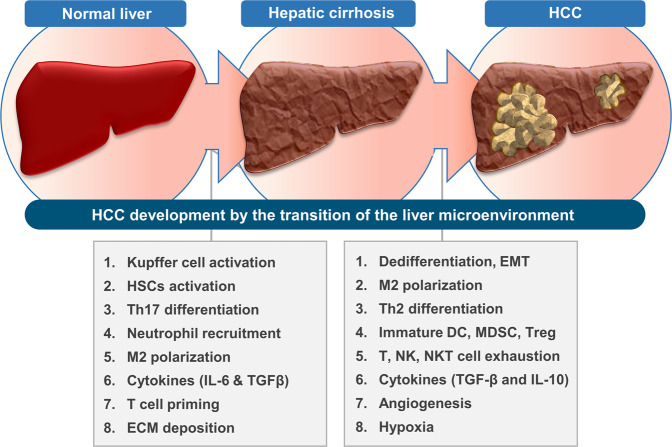
The transition of the liver microenvironment contributes to HCC development. Stimulation by liver injury/damage can induce Kupffer cells to produce proinflammatory cytokines and TGF-β. The migration of immune cells is induced by the proinflammatory response. The proinflammatory immune response can increase Th17 cell differentiation. TGF-β can promote the activation of HSCs, contributing to collagen fiber production and the development of hepatic cirrhosis. In hepatic cirrhosis, the anti-inflammatory response is increased. Increasing Th2 cell differentiation can induce the M2-like transition of macrophages. Activated HSCs can induce the development of MDSCs as well as immature DCs. Increased TGF-β can induce EMT in hepatocytes. Chronic liver inflammation can induce the dedifferentiation of hepatocytes. Hypoxia caused by hepatic cirrhosis and angiogenesis can constitute the protumoral liver microenvironment.

The release of DAMPs and cytokines by damaged hepatocytes induces the transition of the liver microenvironment through immune cell recruitment and proinflammatory responses^13,45,47,168^. Since Kupffer cells are liver-resident macrophages located in the hepatic sinusoidal lumens, they can rapidly respond to danger signals and initiate of liver diseases^71^. In the early phase of liver diseases, Kupffer cell-derived proinflammatory cytokines, including CCL2, IL-1β, IL-6, IL-23, and TNF-α, induce the migration of immune cells and HSCs and contribute to the development of a proinflammatory liver microenvironment^43,45,168^. The proinflammatory microenvironment is accompanied by M1 macrophage polarization, Th17 response, and activation of neutrophils and NK cells to eliminate pathogens, dead cells, early activated HSCs, and tumor cells^43,96,127^ (Fig. 3). However, continuous inflammatory responses in chronic liver diseases not only induce the M2-phenotype transition of macrophages but also induce the activation of HSCs by increasing the expression of profibrogenic cytokines such as IL-6, IL-8, TGF-β, and PDGF-B^35,80,84^. Activated HSCs show a fibroblast-like phenotype and function. They cause hepatic cirrhosis through immune-suppressive responses, an increase in collagen production, an imbalance in MMP/TIMP, and ECM deposition^19,56,92^. In addition, hepatic cirrhosis and activated HSCs can induce immature DC and MDSC development, Th2 cell differentiation, angiogenesis, and hypoxia. They also foster a protumoral liver microenvironment that favors HCC pathogenesis^41,91–93^ (Fig. 3). Moreover, hepatocytes exposed to damage, proinflammatory signaling, the accumulation of genetic alterations, and TGF-β signaling can undergo the EMT or dedifferentiate and be converted to TICs^9,20,37,68^. In the protumoral liver microenvironment of hepatic cirrhosis, the elimination of TICs and HCC cells is inhibited, and HCC development is induced via oncogenic cell proliferation^37,157^.

Kinase signaling pathways play important roles as key regulators throughout the transition of the liver microenvironment and HCC development. Activation of NF-κB signaling is associated with inhibited hepatocyte and HSC death, the upregulation of MMP production in HCC cells, induction of the survival of neutrophils, and CXCL1 production in hepatocytes^46,47,110,119^. Activation of the PI3K/Akt/mTOR pathway can inhibit the apoptosis of hepatocytes. This pathway is also involved in the EMT of hepatocytes, promotion of HCC development, and maintenance of chemotherapy resistance^62,66,169^. The PI3K/Akt pathway is also involved in the activation of HSCs via MAPK^45^. In addition, the activation of MAPK signaling promotes cell proliferation and HCC development^57,66,100^. Activation of the STAT oncogenic pathway promotes Th17 cell development, activates HSCs, causes genetic alteration in hepatocytes, and leads to HCC progression^57,68,70^. Since the regulation of phosphorylation via kinases can be switches that determine the activation and characteristics of cells, kinase signaling pathways have attracted attention as therapeutic targets. A representative example of a tyrosine kinase inhibitor is sorafenib, which has been approved by the FDA as a treatment for advanced HCC, renal cancer, and thyroid cancer^170–172^. Sorafenib blocks various receptor tyrosine kinases (RTKs), such as VEGFR, PDGFR, c-kit, Flt-3, and RET, and suppresses the activation of the MAPK and PI3K/Akt signaling pathways, which play important roles in HCC pathogenesis^173^. Indeed, when the effect of sorafenib was compared with that of a placebo in patients with advanced HCC, sorafenib-treated patients showed an increase in overall median survival by nearly 3 months, in contrast to the effect of placebo on patients^174^. Sorafenib can inhibit the invasion and proliferation of tumor cells via the RAS/MEK/ERK and PI3K/Akt/mTOR pathways^175^. In particular, activation of Akt/mTOR signaling promotes EMT, which is closely associated with tumor invasion^67^. Before the introduction of sorafenib, no effective systemic therapy had been introduced for advanced HCC for ~30 years^174^. Thus, sorafenib is evidence of the therapeutic potential of inhibiting kinase pathways. However, in a study of patients with advanced HCC, the response rate to sorafenib was only 2%, meaning only a partial response was realized, as determined by the response evaluation criteria in solid tumors (RECIST)^174^. The low response rate to sorafenib was also observed in patients with advanced renal cell cancer, in which it was ~2.1%^170^. These ineffective therapeutic outcomes might have occurred because, in addition to hepatocytes, sorafenib acts on other cells, such as immune cells. According to a recent study, sorafenib showed anti-lymphoma activity in lymphoma cell lines by reducing phosphorylation in the PI3K/Akt and MAPK signaling pathways and thus inducing cell death^176^, indicating that sorafenib shows the potential to inhibit the activity of antitumor immune cells. Indeed, high-dose sorafenib showed an immunosuppressive effect on T cells by increasing PD-1 expression^177^. Moreover, Luo et al. suggested that the insufficient therapeutic effect of sorafenib stems from the bidirectional roles of signaling molecules involved in tumorigenesis^136^. Indeed, the loss of hepatic Akt1 in Akt2^-/-^ mice induced HCC pathogenesis^178^. To confront this problem, Luo et al. suggested that targeting factors further downstream and molecules at the terminal end of signaling pathways might lead to more effective outcomes^136^.

Phosphatases regulate the activation of oncogenic pathways induced by various kinases. They are closely related to the development and progression of HCC. Loss of PTEN in hepatocytes induces the activation of Akt and MAPK signaling pathways^134^. PTEN might also be involved in JAK/STAT and NF-κB signaling in macrophages^137^. SHP-1 suppresses the activation of PI3K/Akt signaling and Vav-1 in macrophages^144,146^. In addition, SHP-1 regulates the phosphorylation of Cbl-b and ZAP-70 in T cells and inhibits STAT3 phosphorylation in HSCs^30,149,151^. SHP-2 is involved in the activation of Ras-ERK signaling^157^. The therapeutic impact of sorafenib, a multikinase inhibitor, on HCC suggests the potential of phosphatases as novel targets for inhibiting the development and progression of HCC. However, some phosphatases, such as sorafenib, exhibit bidirectional roles or exert different effects on HCC on the basis of cell type. SHP-2 shows selective suppressive activity on the proliferation of cancer cells^136,156^. PTEN can inhibit the proliferation of hepatocytes and expression of cancer stem cell markers and block HCC development^31,135,136^. Both activation and downregulation of PTEN contribute to M2 polarization in macrophages^33,80^. However, phosphatases are still potential therapeutic targets for treating HCC. For instance, LB-100, a PP2A inhibitor, can enhance the efficacy of conventional anticancer agents^165,166^. In addition, Ssu72 is a very promising HCC treatment target. Ssu72 directly regulates phosphorylation of the downstream transcription factors Rb and HNF4α in hepatocytes, thereby regulating homeostasis and dedifferentiation of hepatocytes^37,160^. Since phosphatases such as Ssu72 can inhibit kinase signaling pathways and regulate transcription, it is clear that phosphatases are potential therapeutic targets for HCC. In relation to HCC progression, regulating the expression or activity of a specific phosphatase in a specific cell may be an efficient therapeutic strategy for HCC.

HCC has a poor prognosis because it is generally diagnosed in a late stage^179^. This issue has been addressed in detail in the American Society of Clinical Oncology (ASCO) guideline for the therapy of advanced HCC^180^. In the early stage of HCC, effective local therapies, including resection and liver transplantation, can be applied^180^. For locally advanced HCC, liver-directed therapies such as transarterial chemoembolization (TACE), bland embolization, and radioembolization can be considered^180^. Indeed, patients with HCC were detected early to show a good prognosis for 5-year survival after treatment^181,182^. However, HCC is commonly found in an advanced or incurable stage. Conventional treatments such as cytotoxic drugs are ineffective in the advanced stage; moreover, they are palliative and do not eliminate the cause of the disease^180,183^. Indeed, the overall survival period for advanced HCC patients is only approximately 6.5–10.7 months^184^. Although systemic therapy for advanced HCC is improved by sorafenib^180^, the response rate is lower than expected^174^. Moreover, an additional option of systemic therapy is not suggested for a decade after the introduction of sorafenib^180,183^. Although combination therapy for advanced HCC has recently been introduced and showed better results^180,183^, it is still important to find novel agents that target the underlying mechanism of tumorigenesis. Targeting broad phosphatases or kinases might lead to side effects or be ineffective because of compensatory pathways. Therefore, targeting the downstream factors in tumor-specific signaling pathways might be a good strategy. In addition, according to the ASCO guidelines, Child–Pugh class A liver disease, in which liver function is preserved, is associated with a good prognosis for HCC^180^. Therefore, chemical agents targeting phosphorylation in signaling pathways might be ineffective in patients with impaired hepatic function. Collectively, inducing overexpression of a phosphatase or kinase that is downregulated in HCC tumor tissue might be the most effective treatment method.
